# Graphene-PbS quantum dot hybrid photodetectors from 200 mm wafer scale processing

**DOI:** 10.1038/s41598-025-96207-z

**Published:** 2025-04-27

**Authors:** Sha Li, Zhenxing Wang, Bianca Robertz, Daniel Neumaier, Oihana Txoperena, Aranzazu Maestre, Amaia Zurutuza, Chris Bower, Ashley Rushton, Yinglin Liu, Chris Harris, Alexander Bessonov, Surama Malik, Mark Allen, Ivonne Medina-Salazar, Tapani Ryhänen, Max C. Lemme

**Affiliations:** 1 AMO GmbH, Otto-Blumenthal-Str. 25, 52074 Aachen, Germany; 2 Chair of Smart Sensor Systems, University of Wuppertal, Lise-Meitner-Str. 13, 42119 Wuppertal, Germany; 3 Graphenea Semiconductor SLU, Donostia, Spain; 4 Emberion Limited, 150-151, Cambridge Science Park, Milton Road, Cambridge, CB4 0GN UK; 5 Emberion Oy, Metsänneidonkuja 8, Espoo, 02130 Finland; 6 Chair of Electronic Devices, RWTH Aachen University, Otto-Blumenthal-Str. 25, 52074 Aachen, Germany

**Keywords:** Electrical and electronic engineering, Graphene, Nanoscale devices

## Abstract

A 200 mm processing platform for the large-scale production of graphene field-effect transistor-quantum dot (GFET-QD) hybrid photodetectors is demonstrated. A comprehensive statistical analysis of the electrical data revealed a high yield (96%) and low variation in the 200 mm scale fabrication. The GFET-QD devices deliver responsivities of 10^5^ to 10^6^ V/W in the wavelength range from 400 to 1800 nm with a response time of 10 ms. The spectral sensitivity compares well to that obtained via similar GFET-QD photodetectors. The device concept enables gate-tunable suppression or enhancement of the photovoltage, which may be exploited for electric shutter operation by toggling between the signal capture and shutter states. The devices show good stability over a wide operation range. Furthermore, an integration solution with complementary metal-oxide-semiconductor technology is presented to realize image-sensor-array chips and a proof-of-concept image system. This work demonstrates the potential for the volume manufacture of infrared photodetectors for a wide range of imaging applications.

**Supplementary Information:**

The online version contains supplementary material available at 10.1038/s41598-025-96207-z.

## Introduction

Graphene possesses outstanding intrinsic properties, such as monoatomic thickness, ultrahigh carrier mobility at room temperature, broadband optical absorption covering the far-infrared (IR) to ultraviolet (UV) range, and an extremely high surface-to-volume ratio. As a result, graphene-based optoelectronic devices are among the ideal candidates for photodetectors^1–3^. In fact, a variety of graphene-based photodetectors have been demonstrated in the laboratory that achieve outstanding performance at the device level, often outperforming their established semiconductor counterparts^4–10^. For example, graphene-based photodetectors have shown responsivities of up to 10^8^ A/W and photoconductive gains of up to 10^8^ electrons per photon in the IR regime^10^. These detectors are potential candidates for extending the bandwidth of silicon (Si) complementary metal-oxide-semiconductor (CMOS)-based photodetectors, which have limited sensitivity at wavelengths longer than 1.13 μm, i.e., with energy below the bandgap of Si. The high IR performance of those detectors was achieved through the hybrid integration of single-layer graphene (SLG) with colloidal quantum dots (QDs) as highly absorbing nanostructures^8,10^. Here, the wavelength of light absorption can be tuned by adjusting the size of the QDs. Furthermore, monolithic integration of a CMOS integrated circuit with graphene field-effect transistor-QD (GFET-QD) photodetectors has also been demonstrated^8^. Although the signal output was several orders of magnitude lower than that of single-pixel reference devices, this early demonstration shows the potential for such devices to provide a low-cost route toward Si technology-based visible to short-wavelength IR (VIS-SWIR) imagers. However, the wet transfer and fabrication processes have not been demonstrated to scale up to wafer size.

A decisive remaining challenge for industrial applications of such GFET-QD photodetectors is the availability of reproducible and wafer-scale “unit processes”^11–13^, which make graphene processing compatible with widely available conventional Si technology fabrication lines. This would be the preferred route toward the commercialization of two-dimensional (2D) materials in microelectronics, as it would require reasonable engineering efforts compared with the development of entirely new production lines from scratch. In addition, application-specific read-out integrated circuits (ROICs) are vital for optimizing the overall system performance, particularly to reduce the noise level. The integration of graphene and 2D materials with CMOS circuits could thus take advantage of the well-established CMOS readout circuitry for signal processing purposes^8^.

Great efforts have been directed at scaling up the synthesis, processing, device integration, and metrology of graphene and 2D materials up to the 300 mm wafer platform^11,12,14–18^. Despite the progress in processing technology, preserving the intrinsic performance of graphene at the device level remains challenging, particularly concerning wafer-scale uniformity and batch-to-batch reproducibility. We have identified the following unit processes that need to be established to realize scalable and high-quality production of high-performing GFET-QD photodetectors: high-quality growth and transfer, patterning with atomic precision, ohmic contacts, adhesion at the interface to graphene, design, and processing of the photosensitive stacks as well as hermetic semiconductor packaging^17,19–23^.

Here, we demonstrate a graphene fabrication process on a 200 mm wafer platform for the large-scale production of GFET-QD photodetectors. This was realized by addressing several of the graphene wafer-scale integration challenges, including monolayer graphene chemical vapor deposition (CVD), transfer, and patterning, which fulfill the requirements for imaging functionalization and large area deposition of the multilayer QD absorber material in an inert atmosphere, as well as methods for encapsulating devices using thin film alumina (Al_2_O_3_) and hermetically sealed semiconductor packages. Such a demonstration elevates the initial concept^8^ to higher technology readiness levels in multiple dimensions, ranging from wafer-scale graphene device statistics to the development of custom CMOS circuits and the implementation of production-ready packaging.

## Results and discussion

### Device design and fabrication

**Fig. 1:**
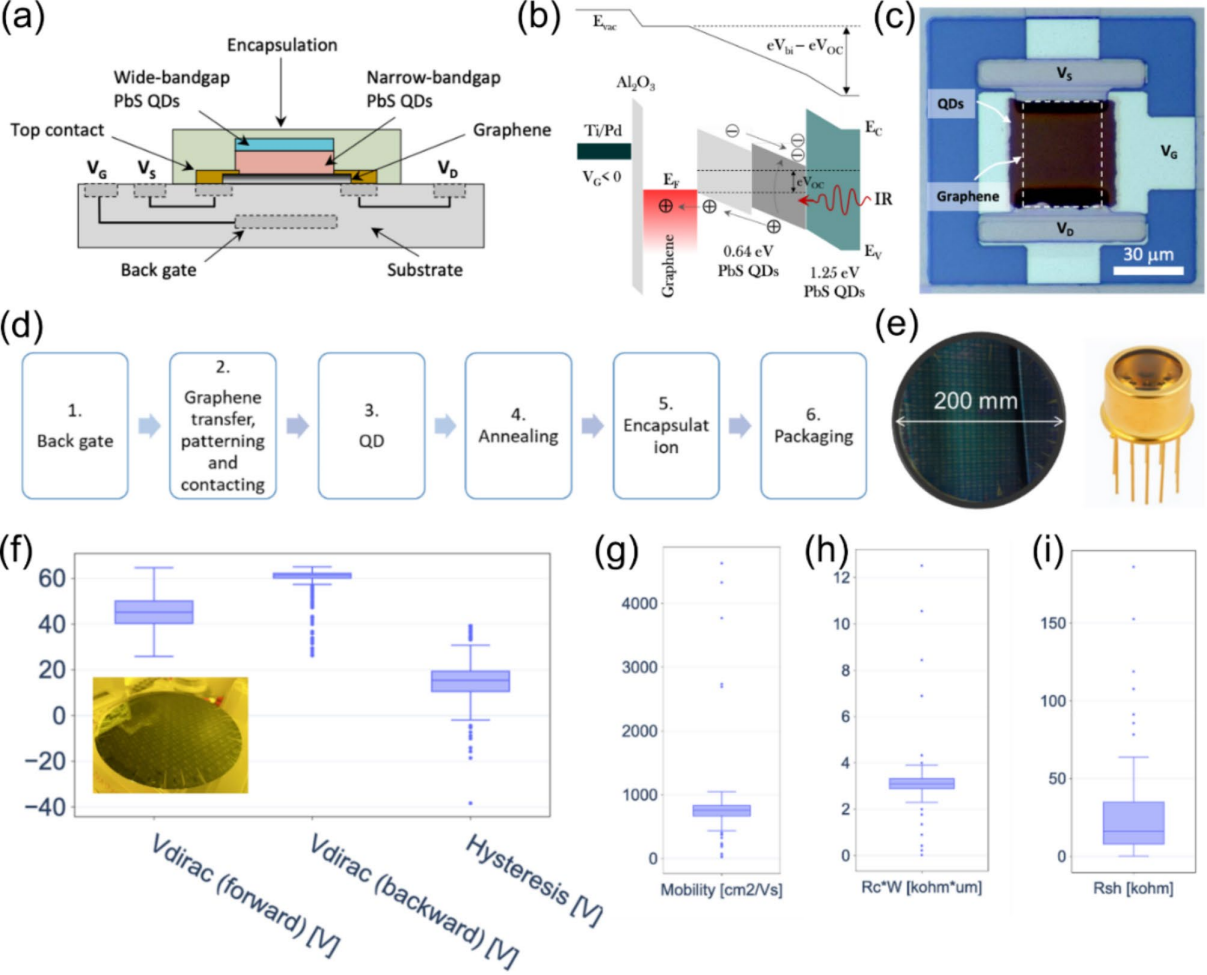
(a) Schematic of the device architecture of the tandem GFET‒QD photodetector. (b) Schematic energy band diagram upon illumination. (c) Top-view optical microscope image of the tandem GFET‒QD photodetector device. (d) Fabrication flow of the GFET-QD photodetector, including the key steps of graphene transfer, patterning, contacting, QD deposition, encapsulation, and packaging. (e) As-fabricated 200 mm wafer (left) and later diced, wire-bonded, and packaged single-pixel photodetector in a hermetically sealed semiconductor package (right). (f–i) 200 mm wafer scale statistics of the electric metrics of encapsulated GFETs. Box-whisker plots of (f) Dirac points and hysteresis, (g) field effect mobility, (h) contact resistance, and (i) sheet resistance of the GFETs. Inset in (f): a photo of the 200 mm wafer after fabrication.

The tandem GFET-QD photodetector device architecture was designed on the basis of graphene active regions covered with two different lead sulfide (PbS) QD layers as the light-sensitive absorber material. The devices include two contacts to the graphene and a buried gate electrode at the back that can be used to tune the device operating point. A schematic cross-section is shown in Fig. 1a.

The working principle of the GFET-QD tandem photodetector can be described based on the energy band diagram illustrated in Fig. 1b. The semiconducting PbS QDs form a heterojunction with the graphene channel. The multilayer absorber comprises a lightly doped or nearly intrinsic PbS QD layer with a narrow bandgap of *E*_g_ = 0.64 eV, which is capped by n-doped PbS QDs with a wide bandgap of *E*_g_ = 1.25 eV. The wide bandgap PbS QD layer is transparent to IR light and acts as a sacrificial layer to prevent the inner QD layers from degrading by moisture and oxygen. In addition, the wide bandgap QDs provide broadband sensitivity that can possibly extend into the visible waveband. If graphene is tuned to be p-doped, the heterojunction resembles a conventional *p-i-n* configuration that is commonly used in QD detectors and photovoltaic applications. The electronic band structure is thus designed to ensure a built-in electric field, which leads to hole transport toward the graphene layer upon optical excitation. The photoexcited charge carrier buildup in the active region upon illumination produces a photocurrent signal in the device, whereas the narrow bandgap of the PbS QDs determines the long wavelength cutoff of the photoresponse. This GFET-QD device is shown in the optical micrograph in Fig. 1c, which includes a local back gate, source and drain terminals and a functionalized QD layer on top of the graphene channel.

The fabrication flow on 200 mm Si wafers is illustrated in Fig. 1d. It consists of the key processing modules of graphene transfer, patterning, contacting, QD functionalization, as well as dicing and packaging. The as-fabricated 200 mm wafer is shown in Fig. 1e (left), and the best devices were packaged into hermetic semiconductor packaging, as shown in Fig. 1e (right), to allow further integration in a camera core. The process flow is based on photolithography, and the details can be found in the “Methods” section.

A separate batch of quality control (QC) wafers was fabricated with GFETs directly encapsulated by 60 nm Al_2_O_3_ without QD functionalization layers. This was done to monitor GFET processing and evaluate the electrical parameters of graphene after device integration because QDs shift the Dirac point via charge doping. In the following section, unless stated otherwise, wafer scale statistics for the electric parameters of graphene were obtained from the QC-wafers, whereas the photodetector parameters were obtained from the photodetector wafers where the GFETs were fully functionalized with QDs.

### Statistics on the wafer scale

We assessed the yield and reproducibility of the fabrication process as well as the suitability of graphene QD functionalization via a combination of noninvasive methods such as optical microscopy, Raman spectroscopy and electrical measurements of test structures. For each processing run, the quality of the monolayer graphene as well as the target parameters for yield, mobility, doping level, and contact resistance were quantified.

Defects and homogeneity across the area of the transferred graphene were investigated via Raman spectroscopy and optical microscopy. Figure S1 shows the inspection protocol, where characterization was carried out on dies covering the edge, middle edge, and center areas (Fig. S1a). Each of the dies contains several devices, and two of the central devices (ID 5-3 and 5-4) were inspected at magnifications of 20 and 100. The image in Fig. S1b shows a single graphene device, where CVD graphene is visible in the central area. The brighter regions of the channel correspond to monolayer graphene, which covers most of the area, whereas the darker regions correspond to multilayer graphene and to wrinkles formed during the cooling of the growth process. This result shows that most of the graphene is in monolayer form and is of suitable quality for the targeted application.

Figure S1c and d show a Raman spectrum and Raman parameters extracted from a representative wafer, with typical graphene fingerprints of the D band at approximately 1350 cm^–1^, the G band at approximately 1582 cm^–1^, and the 2D band at approximately 2700 cm^–1^. The ratio between the intensity of the 2D peak and the G peak (*I*_2D_/*I*_G_*)* indicates the layer number of the graphene. The weak D peak in the Raman spectrum suggests a low number of defects, whereas the *I*_2D_/*I*_G_ ratio of approximately 3 confirms that the graphene is mostly a monolayer.

Figure S1e shows the optical microscopic images of the device at different stages in the process flow.

Automated current-voltage measurements were conducted to extract device characteristics and performance parameters. A photograph of the encapsulated GFETs on a 200 mm wafer is shown in the inset of Fig. 1f. For the 648 devices randomly selected from the 200 mm wafer, a high yield of 96% was achieved. Despite high yield, variation in the performance is still seen in the statistics, which is mainly due to the quality and uniformity of the graphene material. Moreover, the fabrication flow was also found to be stable, with reasonable batch-to-batch reproducibility, confirmed by comparable fabrication yields of 96–98% from three fabrication runs, as summarized in Table S1. The quantified analysis of the graphene electric parameters for the 200 mm wafer is summarized in Fig. 1f, g, h and i, which shows histograms of the mobility, Dirac point (*V*_Dirac_), hysteresis (Δ*V*_Dirac_), contact (*R*_c_), and sheet resistance (*R*_sh_) of the GFETs. Similarly, the analysis was carried out on 150 mm wafers, with a high fabrication yield of 98.4%. A summary of the graphene quality metrics on 150 mm wafers can be found in Table S2, which shows the mobility, *V*_Dirac_, Δ*V*_Dirac_, and *R*_c_, *R*_sh_ of the GFETs. A detailed analysis of the statistics for both the 200 mm and 150 mm wafers can be found in the supporting information.

### Photodetection

The detection performance of the photodetectors was measured and benchmarked with detectors that operate on different detection mechanisms and are made from different materials and geometries. The key parameters of the responsivity (R) are utilized in the following discussion. The responsivity of a photodetector is defined as the ratio of the output photovoltage to the incident light power on the photodetector. It is usually expressed as

1 $$R~\left[ {A/W} \right]=\frac{{{I_{ph}}\left[ A \right]}}{{{P_{in}}~\left[ W \right]}}$$

and

2 $$R~\left[ {V/W} \right]=\frac{{{V_{ph}}\left[ V \right]}}{{{P_{in}}~\left[ W \right]}},$$

where *I*_ph_ is the photocurrent, *V*_ph_ is the photovoltage, and *P*_in_ is the input optical power. This parameter is used to indicate the available output photocurrent or photovoltage of the photodetector for a given incident optical power at a certain wavelength.

**Fig. 2:**
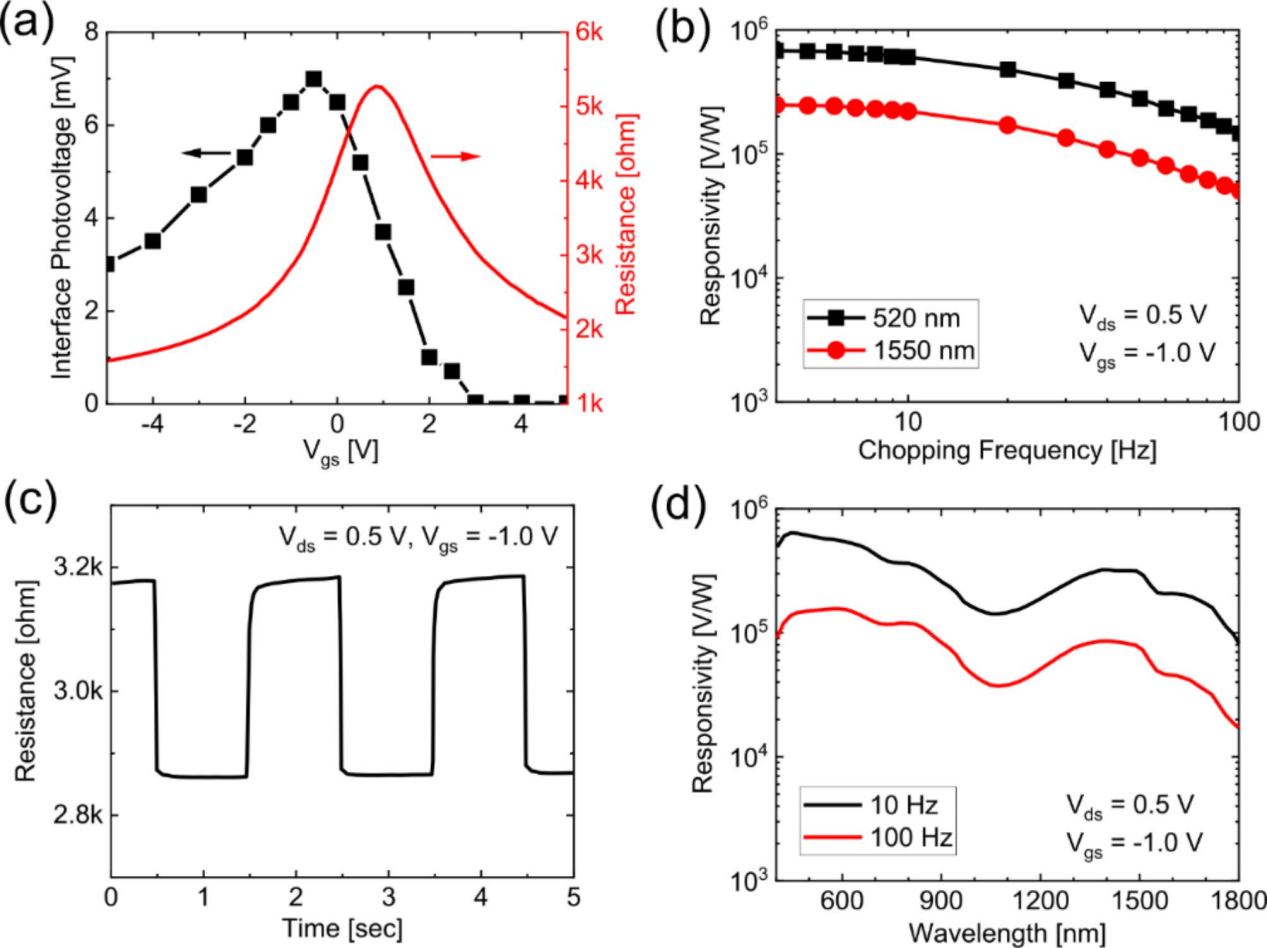
Electro-optical response of the GFET-QD photodetector. (a) Photovoltage emerging at the graphene‒QD junction shown against the *I*_ds_-*V*_gs_ curve of a GFET device (*V*_ds_ = 0.5 V) with an irradiance level < 10^–3^ W/m^2^. (b) Photoresponse curves of the photodetector as a function of chopping frequency, with wavelengths of 520 and 1550 nm (*V*_ds_ = 0.5 V, *V*_gs_ = -1.0 V). (c) Photoswitching behavior of the photodetector toward pulsed light illumination at a wavelength of 1550 nm, light intensity of 0.7 W/m^2^, *V*_gs_ = − 1.0 V and *V*_ds_ = 0.5 V. (d) Photoresponse curves of the photodetector as a function of wavelength, with frequencies of 10 Hz and 100 Hz (*V*_ds_ = 0.5 V, *V*_gs_ = − 1.0 V).

Discrete GFET-QD devices were first measured via a probe station and a parameter analyzer to obtain the transfer characteristics with a local back-gate voltage *V*_gs_ = − 5 to 5 V, as shown by the red curve in Fig. 2a. The electrooptical response (i.e., photovoltage *V*_ph_ as a function of *V*_gs_) was subsequently measured within the same gate sweep range by pulsed illumination using a 520 nm laser diode at 0.5 Hz, as shown in the black curve in Fig. 2a. Comparing the gate-dependent photovoltage at the graphene-QD junction with the transfer characteristics of the GFET-QD device shows a clear influence of the back gate bias, which tunes the depletion layer of the photosensitive QDs by tuning the band alignment between the graphene and QDs. Such behavior is commonly observed for QD-based phototransistors or photodiodes^6,10^. This enables electrically controllable suppression or enhancement of the photovoltage, which can be exploited for realizing electric shutter operation by toggling the back gate between the signal capture and shutter states.

The performance of the GFET-QD photodetectors was further benchmarked by measuring the responsivity of the device as a function of the chopping frequency and wavelength. In both cases, the gate bias was *V*_gs_ = − 1.0 V, with *V*_ds_ = 0.5 V. Figure 2b shows the responsivities for chopping frequencies from 4 to 100 Hz at wavelengths of 520 nm and 1550 nm (black and red, respectively). The responsivity of the detector decreases as the chopping frequency increases since the response time of the device is on the order of a few milliseconds. In the measured frequency range, the responsivity was consistently slightly lower at the larger wavelength of 1550 nm than at 520 nm, although the GFET-QD photodetectors exhibited high responsivities of 10^5^–10^6^ V/W at 100 Hz for both wavelengths. The spectral response was further investigated by measuring the wavelength dependency of the photovoltage for *λ* = 400–1800 nm, as shown in Fig. 2d. The black and red curves represent the responsivity spectra for 10 Hz and 100 Hz, respectively. Comparing the device response at the two frequencies, we note that, consistent with the results of Fig. 2b, the higher frequency delivers a lower responsivity, which indicates that the device frequency response in this case is not especially fast. For *λ* = 400–1800 nm, the responsivity remains rather high, which promises applications in a wider IR range.

The photoswitching behavior of the device was further evaluated by monitoring the time response under an on/off switched light source (Fig. 2c). The device was measured upon pulsed light illumination at a wavelength of 1550 nm and a light intensity of *P* = 0.7 W/m^2^. The corresponding photoresponse stabilized within approximately 1 s.

In summary, the GFET-QD photodetectors operate with a high responsivity of 10^5^− 10^6^ V/W from 400 to 1800 nm with a response time of 10 ms, as well as wavelength sensitivity, comparable to that obtained via similar GFET-PbS QD photodetectors^24,25^. It also operates over a wide wavelength range (400–1800 nm).

**Fig. 3:**
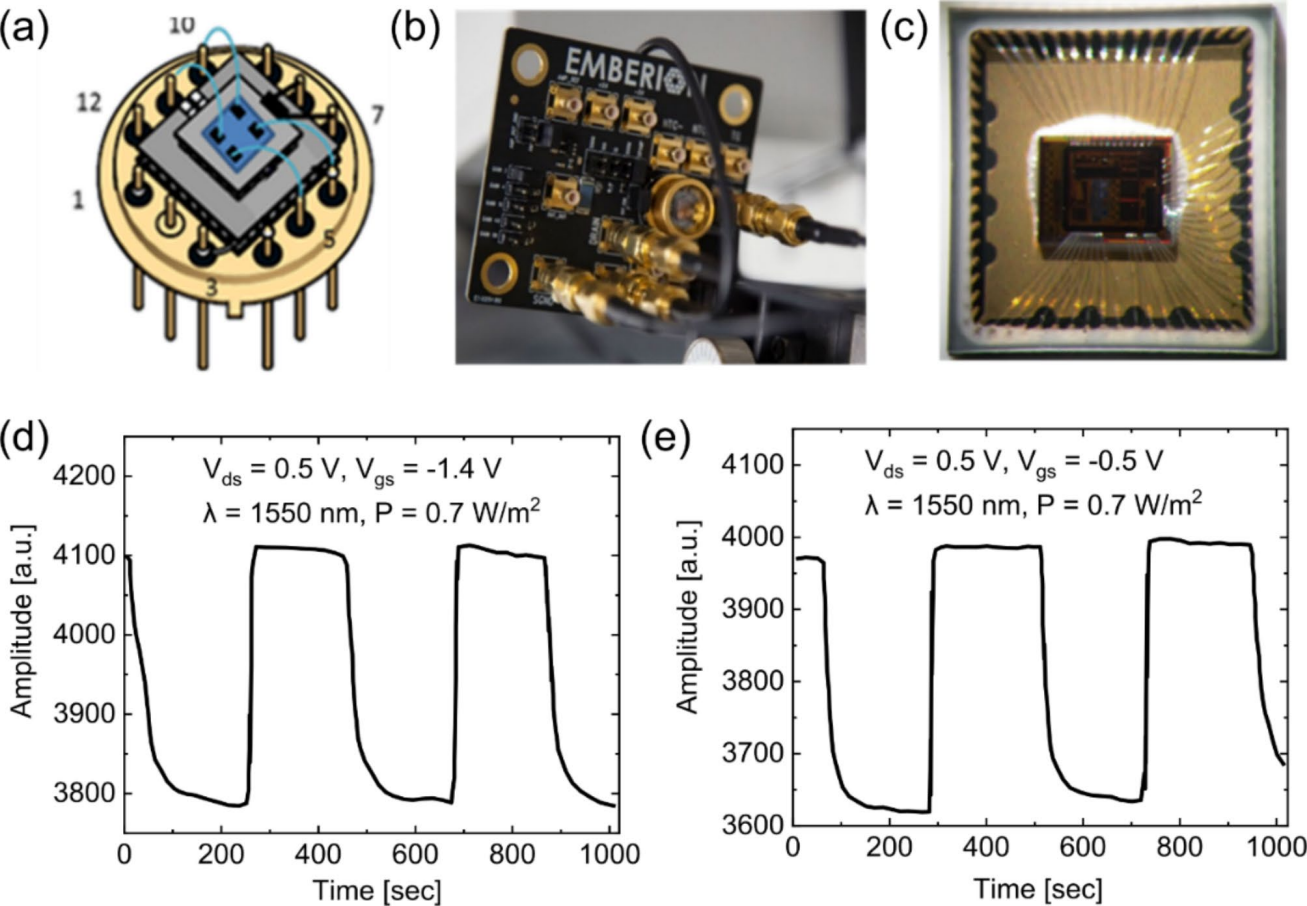
Tandem GFET-QD image sensor. (a) Schematic of a single-pixel photodetector device prior to hermetic sealing into a metal T0-8 semiconductor package with a sapphire window. (b) A single pixel photodetector after hermetic sealing of the sapphire window and connection to a dedicated readout electronics board. (c) Photograph of a few-pixel GFET array fabricated directly on an ASIC and subsequently wire bonded and hermetically sealed into a QFN package. Corresponding photoresponse of the few-pixel array measured through the ASIC at back gate voltages of *V*_gs_ = (d) − 1.4 V and (e) − 0.5 V, *λ* = 1550 nm, *P* = 0.7 W/m^2^, *V*_ds_ = 0.5 V. Units are arbitrary digital values from the DAC output of the ROIC.

Upon successfully developing a 200 mm wafer scale process for GFET-QD photodetectors, we realized image-sensor-array chips and proof-of-concept image systems. To that end, a hermetic semiconductor packaging solution was developed, which allows integration with readout electronics and the creation of an imaging sensor and camera core. This involved designing and testing a semiconductor package to prevent moisture and oxygen from degrading the sensor with the required number of input-output (IO) pins for connection to the application-specific integrated circuit (ASIC) on the parent wafer. Many different packaging options have been investigated with different suppliers, and the first package closure trials have been performed to determine the effects of process parameters on the final device performance. The ROICs needed for data extraction from the sensor devices are not widely available as off-the-shelf items, as the GFET-based sensors often require specific protocols and voltage levels to obtain the best performance, so we developed a modular readout architecture for high-speed and low-noise applications being targeted. In first-generation single-pixel detectors, the ROIC was built on a separate printed circuit board (PCB) connected to the IR detector (Fig. 3a and b). In later device generations, as in the case of 512-pixel linear array detectors and video graphics array (VGA) imagers, the ROIC was integrated directly with the detector array sensor die. An example of a few-pixel GFET array (16 pixels) in a quad flat no leads (QFN) package, which is fabricated via the same process but on a smaller wafer piece, is shown in Fig. 3c. In this case, the GFET-based sensors were fabricated directly on top of the planarized ASIC bare die. Once the fabrication steps were complete, a QFN package was used that had 64 pin-out connections. The detector die was bonded to the package, and then the pads on the detector die were wire bonded to the package pins before sealing in an inert atmosphere. The photoresponse of a few-pixel array fabricated directly on the ASIC was measured through the ASIC at back gate voltages of *V*_gs_ = − 1.4 V and − 0.5 V at a wavelength of 1550 nm and a light intensity of 0.7 W/m^2^. The photocurrents in Fig. 3d and e are plotted in arbitrary units that originate from digital values directly taken from the digital-to-analog converter (DAC) output of the ROIC.

## Conclusions

We have demonstrated the successful integration of QD-functionalized GFETs with CVD graphene on a 200 mm CMOS wafer platform, which provides a route for volume manufacturing of IR photodetectors. These sensors have many imaging applications, ranging from surveillance, search and rescue, and vehicle safety to improved sorting of food and food packaging to reduce their environmental impact. This work illustrates the level of maturity of electro-optical devices based on monolayer CVD graphene as well as the current status regarding the main challenges associated with the growth, transfer, and patterning of graphene. The results further support the successful large-area deposition of multilayer QD absorber materials in an inert atmosphere, including methods to encapsulate devices via thin-film Al_2_O_3_ and hermetically sealed semiconductor packages. In summary, we have shown potential paths toward manufacturing graphene-based devices at the back end of the line and their packaging into a technology demonstrator for IR sensing.

## Methods

### Fabrication of the photodetector

High-quality graphene was grown in a BM Pro 2 × 8” CVD furnace from Aixtron Ltd., using methane (CH_4_) as the precursor and copper (Cu, 200 mm × 200 mm foil) as the catalyst. A semi-dry transfer was carried out using a sacrificial polymeric support (polymethyl methacrylate, PMMA, prepared by spin coating) placed on the graphene surface, and the Cu foil was etched away via an ammonium persulfate (APS) etchant. After the bottom surface of the graphene was rinsed with DI water, it was dried and stamped on the target substrate via uniaxial pressure. Finally, the polymer was removed via solvents. A similar process has been used to establish wafer-scale integration of graphene on a 300 mm pilot wafer, which proves the compatibility of such transfer process with CMOS foundry environment^26^.

Local back-gated graphene GFETs were fabricated on 150 mm and 200 mm Si wafers with 90 nm silicon dioxide (SiO_2_) via standard CMOS-compatible photolithography technology. First, the titanium/palladium (Ti/Pd) local back gate with a thickness of 5/40 nm was evaporated onto the substrate, followed by a standard metal lift-off process. A 75 nm thick Al_2_O_3_ layer was subsequently deposited via the atomic layer deposition (ALD) method to form the dielectric layer. Afterward, the bottom contact of 30 nm Pd was evaporated. After transfer, the graphene was patterned via reactive ion etching (RIE) in an oxygen plasma. Then, a final layer of 40 nm Pd was evaporated onto the substrate as the top contact.

The QDs were obtained from Quantum Solutions and had diameters on the order of 6–7 nm according to the data sheet. QD deposition was performed by spin-coating a total of 12 layers of PbS QDs via layer-by-layer ligand replacement in an inert atmosphere glovebox to minimize exposure to water and oxygen, followed by encapsulation with an aluminum oxide layer deposited via an ALD tool. The oleic acid ligands were replaced with ethanedithiol (EDT) or benzene dithiol (BDT) by in-situ ligand replacement. There are two different sizes of QDs with two different ligands, resulting in a 4-4-4 absorber-type arrangement (4 coatings of QDs with a 0.64 eV bandgap, another 4 coatings of QDs with a 0.64 eV bandgap, and finally, 4 coatings of QDs with a 1.25 eV bandgap), with an overall thickness on the order of 300 nm. Figure S2a–d show the 200 mm wafer before and after 4-, 8-, and 12-layer QD deposition, respectively. The absorber layer was then removed everywhere except on the graphene channels of the GFETs via lithographic patterning and a solution-based etching process (concentrated HCl and HI acid solutions). The devices were finally encapsulated with Al_2_O_3_ via an ALD process. Figure S2e shows two quadrants of the 200 mm wafer after dicing, lithographic patterning, and ALD encapsulation. The absorber layers are well aligned on the graphene channels of the GFETs (close-up view in Fig. S2f). After measurements with a probe station and a laser diode for illumination, the best devices were diced, wire bonded, and packaged into hermetic semiconductor packaging to allow further integration with readout electronics to create an imaging sensor and camera core.

### Characterization

Graphene on wafer samples was optically inspected under a Nikon LV100 microscope in bright field mode, and Raman spectra were acquired by a confocal Raman microscope from WITec (Alpha 300) at a laser wavelength of 532 nm.

### Electrical measurements and parameter extraction

All electrical measurements were performed at room temperature under ambient atmosphere in a two-point probe station with a semiconductor parameter analyzer (HP 4156B). The transfer characteristics *I*_ds_–*V*_gs_ of the GFETs were measured with a *V*_ds_ of 100 mV and a *V*_gs_ of (− 5, 5) V.

The TLM data was used to extract the contact and sheet resistances. Local back-gated GFETs with channel lengths *L* varying from 9 to 89 μm in steps of 10 μm and a channel width *W* of 19 μm are fabricated. The total resistance *R*_t_ consists of the channel resistance from graphene, and the contact resistance from the graphene/metal junction can be expressed as *R*_t_(*L*) = *R*_sh_*L*/*W* + 2*R*_c_/*W*. The total resistance (*R*_t_) of each neighboring contact pair was measured as a function of the back-gate voltage (*V*_gs_), with a *V*_ds_ of 100 mV. All devices show the typical behavior of GFETs, where the resistance maximum indicates the charge neutrality point (*V*_Dirac_). By linear fitting the *R*_t_ values of GFETs with different *L* values under the same net gate voltage (*V*_gs_-*V*_Dirac_), *R*_sh_ and *R*_c_ at this gate voltage can be extracted from the slope and intercept, respectively. Gate voltage-dependent *R*_sh_ and *R*_c_ curves can also be obtained.

Mobility was extracted by the field-effect mobility model, also called direct DTM^27–29^. Using the gate voltage-dependent transconductance of the GFETs, the field-effect mobility *µ* is calculated as follows: *µ* = *g*_m_*L*/(*WV*_ds_*C*_gs_), where transconductance *g*_m_ = ∂*I*_ds_/∂*V*_gs_, the graphene channel length *L* and width *W*, the drain voltage *V*_ds_ = 100 mV, and the gate capacitance *C*_gs_ = *εε*_0_/*t*_ox_, with *t*_ox_ = 90 nm, *ε* = 3.9 (SiO_2_) and *ε*_0_ = 8.85 × 10^−12^ F/m.

The electro-optical response of the GFETs was measured in an MPI probe station with a Keithley BF1500 parameter analyzer to obtain the transfer curves of the GFET-QDs as a function of the back-gate voltage, followed by pulsed illumination via 520-nm and 1550-nm laser diodes at 0.5 Hz. The photovoltage and the voltage response were calculated based on the resistance between the source and drain of the GFET. Here we use a similar measurement setup as in Reference^24^. A photo depicting the setup is shown in Fig. S3.

## Electronic supplementary material

Below is the link to the electronic supplementary material.

Supplementary Material 1

## Data Availability

The data supporting this study are available when reasonably requested from the corresponding author.
