# Supplementary material for: Graphene-PbS quantum dot hybrid photodetectors from 200 mm wafer scale processing
Source: Sci Rep. 2025 Apr 27;15:14706. doi: 10.1038/s41598-025-96207-z (PMC12034788; doi:10.1038/s41598-025-96207-z)
Supplement: Supplementary file 1 — Supplementary Material 1 [file 41598_2025_96207_MOESM1_ESM.docx]

Supporting Information for

**Graphene-PbS Quantum Dot Hybrid Photodetectors from 200 mm Wafer Scale Processing**

*Sha Li,^1^ Zhenxing Wang,^1,*^ Bianca Robertz,^1^ Daniel Neumaier,^1,2^ Oihana Txoperena,^3^ Aranzazu Maestre,^3^ Amaia Zurutuza,^3^ Chris Bower,^4^ Ashley Rushton,^4^ Yinglin Liu,^4^ Chris Harris,^4^ Alexander Bessonov,^4^ Surama Malik,^4^ Mark Allen,^4^ Ivonne Medina-Salazar,^4^ Tapani Ryhänen,^5^ Max C. Lemme^1,6,*^*

^1^ AMO GmbH, Otto-Blumenthal-Str. 25, 52074 Aachen, Germany

^2^ University of Wuppertal, Chair of Smart Sensor Systems, Lise-Meitner-Str. 13, 42119 Wuppertal, Germany

^3^ Graphenea Semiconductor SLU. Donostia, Spain

^4^ Emberion Limited, 150-151, Cambridge Science Park, Milton Road, Cambridge, CB4 0GN, UK

^5^ Emberion Oy, Metsänneidonkuja 8, 02130 Espoo, Finland

^6^ RWTH Aachen University, Chair of Electronic Devices, Otto-Blumenthal-Str. 2, 52074 Aachen, Germany

^*^ Correspondence: [wang@amo.de](mailto:wang@amo.de), [lemme@amo.de](mailto:lemme@amo.de)

- **Analysis of statistics for 200 mm wafers**

Automated current-voltage measurements were conducted to extract device characteristics and performance parameters (drain current *I*_ds_ vs. gate voltage *V*_gs_). The measurements were taken under ambient conditions at room temperature, with a source-drain voltage of *V*_ds_= 100 mV and *V*_gs_ swept from -5 to 5 V. A device is categorized as “defective” when the gate leakage (*I*_gs_) exceeds 100 nA, a short circuit channel (*I*_ds_ > 5 mA) or an open circuit channel (*I*_ds_ < 100 nA) is detected, or when the drain current *I*_ds_ modulation as a function of *V*_gs_ is < 10%. Among the 648 devices randomly selected from the 200 mm wafer, 624 were categorized as functional, and 24 were categorized as defective (short- or open-circuit channels and/or leakage), corresponding to a yield of 96%. This high yield demonstrates successful wafer-scale graphene transfer and includes process steps such as the fabrication of graphene contacts and gate dielectric deposition between the gate and graphene channels. The fabrication flow was also found to be stable, with reasonable batch-to-batch reproducibility, confirmed by comparable fabrication yields of 96–98% and a resulting field effect mobility of 720–1000 cm^2^/V∙s from three fabrication runs, as summarized in **Table S1**.

The quantified analysis of the graphene electric parameters is based on mobility, the doping level, the modulation of *I*_ds_ with *V*_gs_, contact (*R*_c_), and the sheet resistance (*R*_sh_). These were extracted from local back-gated transfer length method (TLM) structures with channel lengths L varying from 9 to 89 *μ*m in steps of 10 *µ*m and a fixed channel width *W* of 19 *μ*m. *R*_c_ and *R*_sh_ were extracted via TLM; mobilities were calculated through the field-effect mobility model via the direct transconductance method (DTM) (see Methods for details on electrical data extraction). The 200 mm wafer scale statistics are summarized in **Figures 1f-1i**, which show histograms of the mobility, Dirac point, hysteresis, and *R*_c_, *R*_sh_ of the GFETs. The mobility of the measured devices is 719 ± 172 cm^2^/V∙s. Although higher numbers have been routinely achieved for CVD graphene devices in the literature, these values are not critical for the application targeted here. Moreover, the devices are heavily p-doped, which is reflected by the Dirac points centered around *V*_gs_ ~ 40 V. Possible reasons are the strain and residue introduced by the graphene transfer ^1^ and fabrication process ^2^, as well as the fact that the devices were measured in ambient air, which is known to lead to p-doping ^3^. We further observed a rather high hysteresis in the transfer characteristics, which can be attributed to water molecules that act as charge-trapping centers ^4^. The water molecules are likely due to exposure of the as-transferred graphene to ambient air and moisture while transporting the wafers between different sites.

- **Analysis of statistics for 150 mm wafer**

For device yield and electric metrics analysis, fully automated transfer characteristics measurement (*I*_ds_-*V*_gs_) was performed on the as-fabricated QC wafers under ambient conditions at room temperature, with *V*_ds_ at 100 mV and *V*_gs_ at (-5, 5) V. Out of the 440 devices randomly picked from a 150 mm wafer, 433 were measured as functional and seven defective (short or open circuit channels, gate leakage, or low modulation), corresponding to a yield of 98.4%.

For the quantified analysis of graphene electric metrics, the mobility, doping level, modulation, contact (*R*_c_), and sheet resistance (*R*_sh_) were probed from local back-gated transfer length method (TLM) structures (channel length L varying from 9 to 89 *μ*m in steps of 10 *µ*m and channel width *W* of 19 *μ*m). *R*_c_ and *R*_sh_ were extracted via the TLM; mobility was calculated via the field-effect mobility model of the direct transconductance method (DTM) (see Methods section for details on electrical data extraction). A summary of the graphene quality metrics on 150 mm wafers can be found in **Table S2**, which shows the mobility, Dirac point, hysteresis, and *R*_c_, *R*_sh_ of the GFETs. The mobility of the measured devices across the whole wafer is 849±351 cm^2^/V∙s, and the hysteresis is 0.5 V.

- **Details of the electro-optical measurements**

Detailed electro-optical measurements of the as-fabricated 200 mm wafers can be found in **Figure S4**. In general, the transfer curves of the underlying GFETs were good compared with those of earlier samples, with Dirac point voltages of approximately 1–2 V and low levels of hysteresis. It is impossible to determine whether the hysteresis originated in the underlying GFET or was a result of the absorber stack since the GFET transfer curves cannot be reliably measured before ALD encapsulation. A selection of GFET-QD transfer curves along with the photoresponse measured at *V*_gs_ = 0 V and *V*_ds_ = 0.5 V, and 24 W/m^2^ IR light at a wavelength of 1550 nm pulsed at 0.5 Hz are shown in **Figure S4b**. Typically, the charge mobility measured for both N- and P-branches is approximately 2000 cm^2^/V∙s, with a hysteresis of 0.2 V and a Dirac voltage of 1 V. When illuminated with 1550 nm IR light, the average change in channel resistance between dark and illuminated levels is ~ 3.6% with an SNR of approximately 177, with a photocurrent on the order of 6 *µ*A.


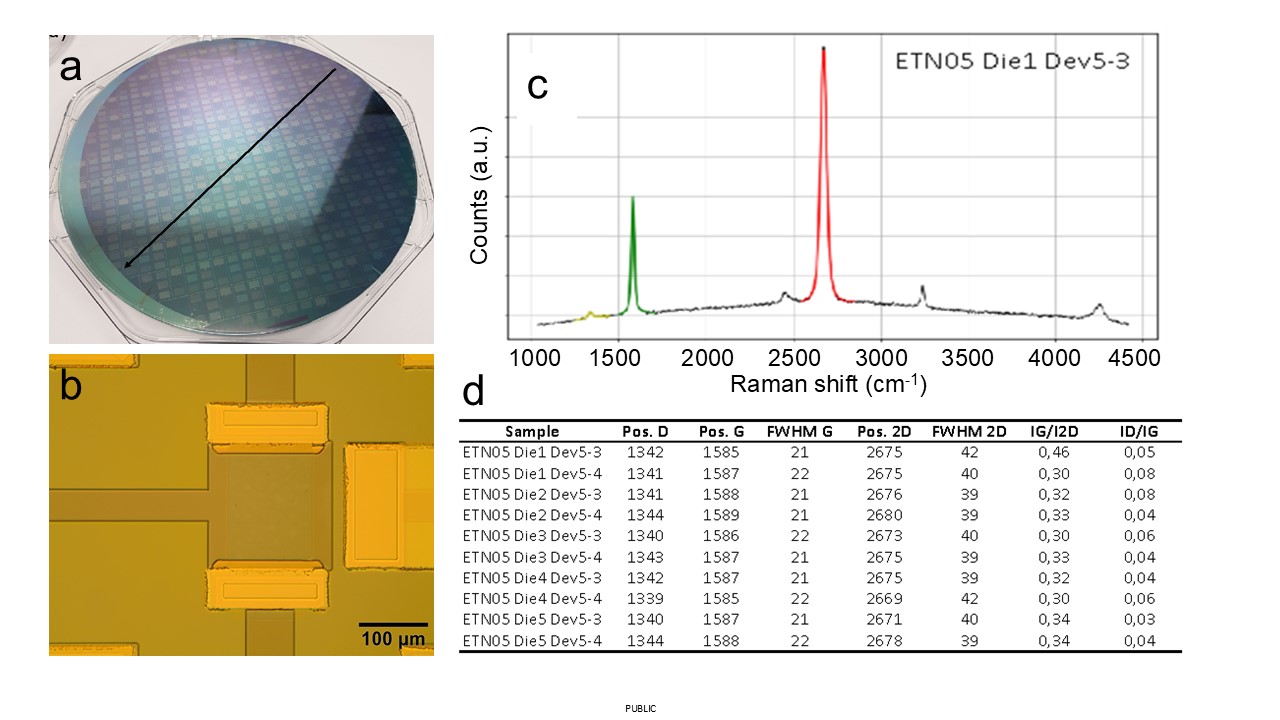


e


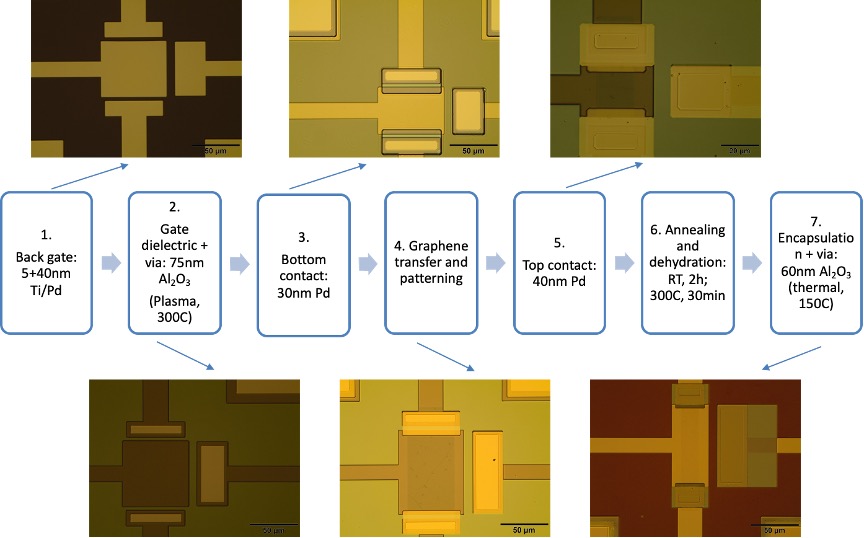


**Figure S1**. (a) 200 mm test wafer, where diagonal dies are routinely inspected. (b) Optical image of a representative device. (c) Typical Raman spectrum and d) Raman parameters extracted for a representative graphene-on-wafer sample. Raman spectra were used to assess the graphene quality. The D band is related to disorder and defects in graphene, whereas the G band arises from sp^2^ carbon networks, and the 2D band corresponds to the overtone of the D band. (e) The optical microscopic images of the device at different stages in the process flow.


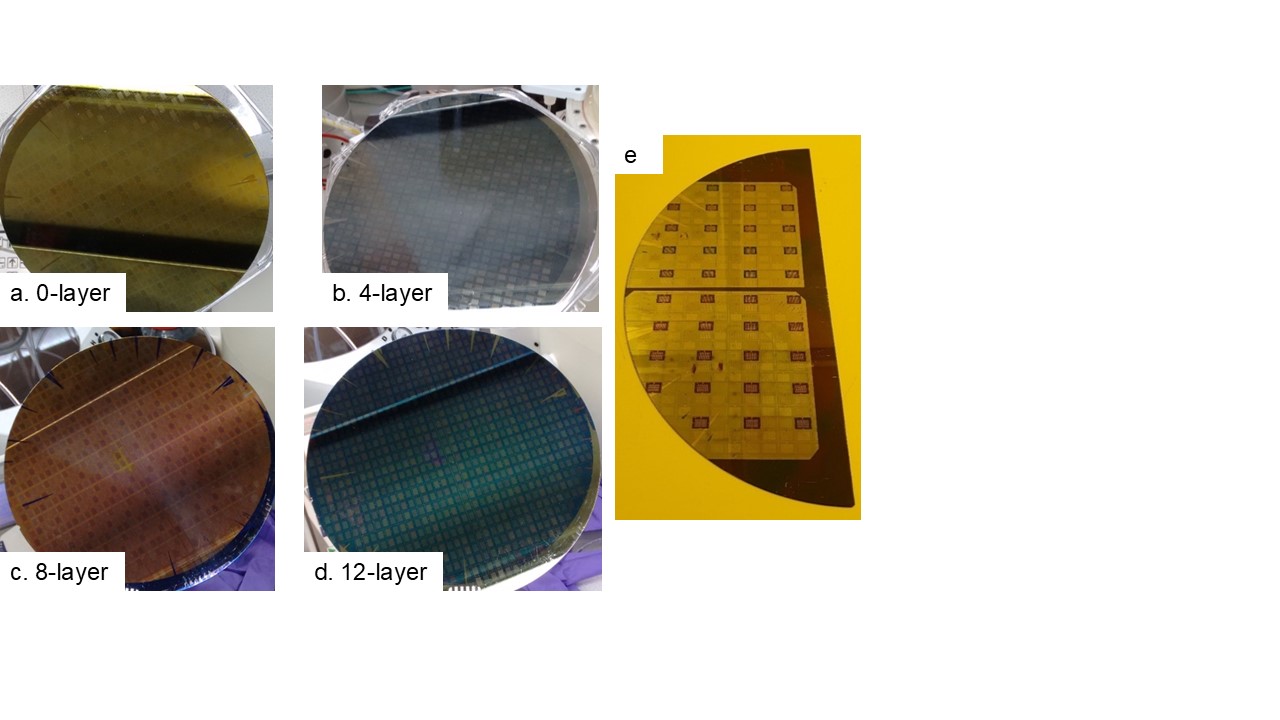


**Figure S2**. Deposition of PbS QD layers on a 200 mm wafer: (a) Wafer with a 200 mm graphene layer, (b) after deposition of the first four layers of QDs, (c) after deposition of eight layers, and (d) after deposition of all twelve layers of the absorber stack. 200 mm wafer after dicing and lithographic patterning and solution etching to remove the absorber layer, except in the graphene channel regions (e).


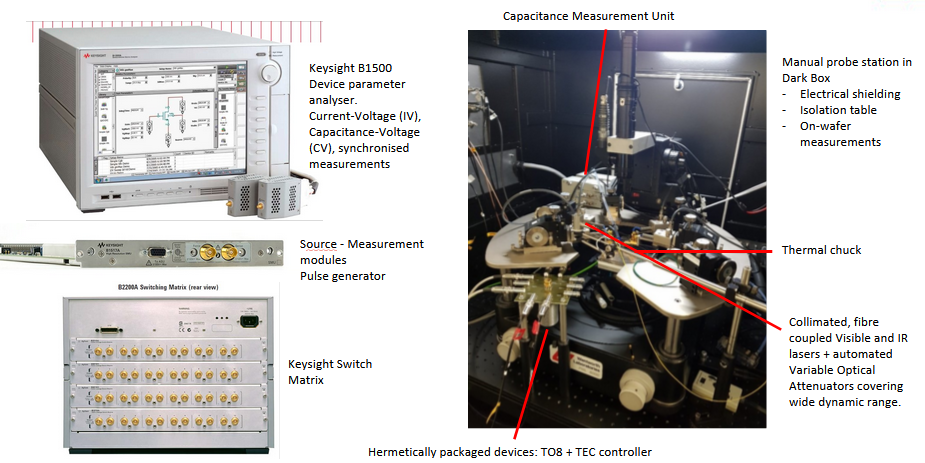


**Figure S3**. The setup for the electro-optical measurements.


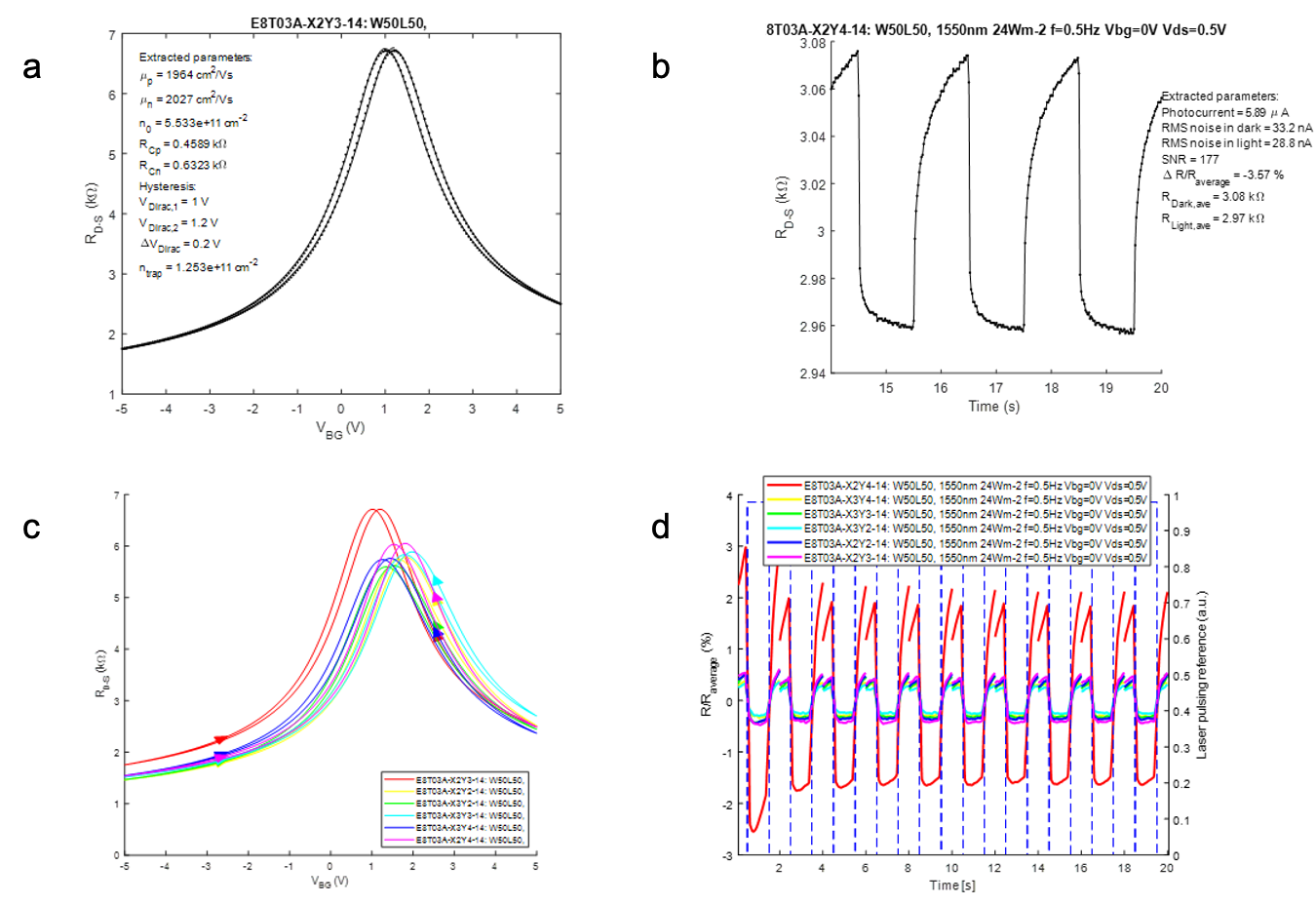


**Figure S4**. (a) Transfer curve for the QD-functionalized GFET, showing a Dirac point of ~ 1 V with 0.2 V hysteresis. (b) Photoresponse at a light power of 24 W/m^2^ and a wavelength of 1550 nm at 0.5 Hz using V_gs_ = 0 V and V_ds_ = 0.5 V. (c) Transfer curves for a small array of devices and (d) the corresponding photoresponse at 1550 nm at a light power of 24 W/m^2^ using V_gs_ = 0 V and V_ds_ = 0.5 V.

**Table S1.** Fabrication results of encapsulated GFETs from different batches.

| **Wafer No.** | 01 | 02 | 03 |
| --- | --- | --- | --- |
| **Wafer scale** | 150 mm | 150 mm | 200 mm |
| **Yield** | 98.4% | 97.5% | 96% |
| **Mobility (cm^2^/V∙s)** | 849 ± 351 | 1055 ± 580 | 719 ± 172 |

**Table S2.** Quality metrics of encapsulated GFETs on a 150 mm wafer scale. (*Some of the defective devices fall into multiple non-working categories)

| **Wafer No.** | 01 |
| --- | --- |
| **Graphene transfer method** | Semi-dry |
| **Yield** | 433/440 = 98.4%*   - Gate leakage (I_gs_ > 100 nA): 4 - No contact (I_ds_ < 100 nA): 6 - Channel short (I_ds_ > 5mA): 0 - No modulation (< 10%): 4 |
| **Mobility (cm^2^/V∙s)** | 849 ± 351 |
| ***V*_Dirac_ for forward scan (V)** | 5.1 ± 0.9 |
| ***V*_Dirac_ for backward scan (V)** | 5.7 ± 0.9 |
| **∆*V*_Dirac_ (V)** | 0.5 ± 0.7 |
| ***R*_c_∙*W* (kΩ*µm)** | 19 ± 20 |
| ***R*_sh_ (kΩ/□)** | 6.7 ± 1.5 |
| **Modulation (*I*_ds,max_/*I*_ds,min_)** | 4 ± 0.9 |

**References**

1. Inhomogeneous strain and doping of transferred CVD-grown graphene | SpringerLink. https://link.springer.com/article/10.1007/s12598-021-01912-z.

2. Choi, A. *et al.* Residue-free photolithographic patterning of graphene. *Chemical Engineering Journal* **429**, 132504 (2022).

3. Szafranek, B. N., Schall, D., Otto, M., Neumaier, D. & Kurz, H. High On/Off Ratios in Bilayer Graphene Field Effect Transistors Realized by Surface Dopants. *Nano Letters* **11**, 2640–2643 (2011).

4. Smith, A. D. *et al.* Resistive graphene humidity sensors with rapid and direct electrical readout. *Nanoscale* **7**, 19099–19109 (2015).
